# Measurement of Microcystin -LR in Water Samples Using Improved HPLC Method

**DOI:** 10.5539/gjhs.v7n2p66

**Published:** 2014-09-28

**Authors:** Hamid Reza Shamsollahi, Mahmood Alimohammadi, Ramin Nabizadeh, Shahrokh Nazmara, Amir Hossein Mahvi

**Affiliations:** 1 School of Public Health, Tehran University of Medical Sciences, Tehran, Iran; 2 Center for Solid Waste Research, Institute for Environmental Research, Tehran University of Medical Sciences, Tehran, Iran

**Keywords:** microcystins, cyanobacteria, limit of detection, HPLC

## Abstract

Microcystins are a group of toxic compounds produced by freshwater cyanobacteria and cause diseases. World Health Organization has recommended a concentration of 1 µg/l for Microcystin-LR (MC-LR) in potable water as guideline value. The high performance liquid chromatography (HPLC) followed by C_18_ analytical column and ultra violet detector for detection of MC-LR. In this regard, 5 different concentrations of MC-LR solutions were injected into HPLC. MC-LR was detected in 5.33 minute retention time and Calibration curve was achieved with R^2^=0.988. Detection limit for this method was obtained by using acetonitrile solutions (32% and 55%) as a gradient run and a high silanol activity column equal to 0.02 µg/mL. Despite no acidic organic modifier being used in the mixture of solvents, the sensitivity of this method was appropriate for detection of MC-LR. Because of short retention time, reduction in number of solvents and high resolution and suitable sensitivity, this method is affordable and is fast for detection and determination of MC-LR in potable water.

## 1. Introduction

Safe drinking water is very important as a matter of health point of view in a community. Microcystins (MCs) are a group of toxic compounds being produced by freshwater cyanobacteria, such as *Anabena*, *Nostoc*, *microsistis* and *Oscillatoria*. MCs are cyclic heptapeptides that have a general structure including D- Alanine (D-Ala), X, D- erythro-β- methylaspartic acid (D- MeAsp), Z, unique C_20_ β- amino acid,(2S,3S,8S,9S)-3- amino-9- methoxy-2,6,8-trimethyl-10-phenyldeca-4(E), 6(E)-dienoic acid (Adda), D-Glutamic acid (D-Glu), and methyldehydroalanine (Mdha). The X and Z types are two variable L-Amino acid in position 2 and 4 of cyclic structure (Dawson, 1998, Edwards, Christine, Linda, & Lawton, 2010; Lawton, Linda, & Christine Edwards, 2001; Sangolkar, Lalita et al., 2006).

Microcystin-LR (MC-LR) is one of the highest toxic MCs that contain Lucien (L) and Arginine (R). MC-LR can cause acute and chronic toxicity in human and animals which inhibits the enzymatic function of PP1 and PP2A enzymes. Exposure to high levels of MCs can cause hepatocyte necrosis, hemorrhage, and finally death. In addition, long term exposure to low levels of toxin causes tumor promotion in the body (Lawton, Linda, & Christine Edwards, 2001; Sangolkar, Lalita et al., 2006).

The guideline value recommended by World Health Organization (W.H.O) for MC-LR concentration in potable water is 1 µg/L. Determination methods of MCs in water samples need to be very sensitive, due mainly to its trace amounts in water (Edwards, Christine, Linda, & Lawton, 2010; Sangolkar, Lalita et al., 2006).

Several methods such as bioassay, enzyme-linked immunosorbent assay (ELISA) and Liquid Chromatography (LC) methods with mass spectrometric or ultra violet (UV) detector have been used for MCs detection (An & Wayne, 1994; Harada et al., 2004; Lawton, & Edwards, 2001; Lawton et al., 1999, Sangolkar et al., 2006; Spoof et al., 2003). High Performance liquid Chromatography (HPLC) with UV detector is an acceptable sensitive method for identification and quantification of MC-LR (Rivasseau, Corinne et al., 1998).

Limit of detection (LODs) by UV detector in determination of MCs is reported to be below 1 µg/L, which is suitable for detecting trace amounts of MCs in water samples (Dawson, 1998; Rapala et al., 2002). Achieving good separation and sensitivity of an HPLC method is related to several parameters such as mobile phase components, HPLC condition, including temperature, flow rate, and column features, e.g. length, silanol activity, and materiel of stationary phase (Mayer & Veronika, 2004). In many investigations, several solvents have been applied as the mobile phase in different percentages of organic solvent and water in detection of MC-LR (Chow et al., 1999; Sangolkar et al., 2006). For achieving a good separation and high resolution, many investigations have been applied using tri- fluoroacetic acid (TFA) as acidic organic modifier in the mixture of mobile phase (Aguete et al., 2001; Lawrence, James, & Cathie Menard, 2001; Purdie et al., 2009, Song et al., 2009).

In the present study, MC-LR detection was performed by a mixture of water and acetonitrile (ACN) with no TFA as acidic organic modifier. Simplification in mixture of solvents was performed to achieve an economical, fast and sufficiently accurate method for detection of MC-LR. Since, MC-LR is more hydrophilic than other MCs variants (Vesterkvist, Pia, Jussi, & Meriluoto, 2003); therefore, reversed phase liquid chromatography is a much suitable method for detecting MC-LR (Mayer & Veronika, 2004). The main objective of the present study was to detect MC-LR levels in water samples based on the polarity of solvents and column silanol activity in achieving both shorter retention time and good sensitivity to set up an affordable and fast analytical method for detection and determination of MC-LR in potable waters.

## 2. Material and Methods

### 2.1 Chemicals

MC-LR standard obtained from ENZO Life Science (England). Methanol, Acetonitrile and distillated water were purchased from CALEDON laboratory chemicals, Ontario, Canada. All the reagents were HPLC grade. MC-LR stock solution was prepared by mixing MC-LR standard with methanol, and then it was centrifuged in 2000 RPM for 15 min. MC-LR standard solutions were prepared in different concentrations by adding desired volumes of stock solution to methanol. The prepared standard solutions were 0, 20, 40, 80 and 100 µg/L of concentrations. For prevention of MC-LR losses, all the standard solutions were made in glass containers (Hyenstrand et al., 2001).

### 2.2 HPLC Analysis

Applied analytical column was C18, ODS A, Tracer Excel (25 × 0.46 cm) with 5 µm particle size, purchased from TEKNOKROMA, Barcelona, Spain. Volume of injected samples were 100 µL. HPLC system was Knauer, (Germany) equipped by UV array detector (Knauer, Germany) set at 238 nm for detection of MC-LR. The column temperature was 30°C. Two mobile phases were applied including: A) with 32% acetonitrile and B) with 55% acetonitrile (v/v). Applied analytical procedure was gradient run as follows: 0 min and 100% A, 12 min and 50% A, 15 min and 100% B, 25 min and 100% A, 60 min and 100% B. The flow rate was kept in the range of 1 mL/min.

## 3. Result

Calibration curve was achieved using standard solutions by 5 different concentrations of MC-LR in the range of 0.02 - 1 µg/mL. The calibration curve characters are shown in Table 1.

**Table 1:** data Achieved from MC-LR Calibration curve with HPLC system

Curve characters	Values
Regression equation	y = 0.005 x
R^2^	0.988
RF stDev	0.00057
RF % RSD	10.563
Average RF	0.0054
Detection limit(µ/ml^-1^, S/N =5)	0.02

**Figure 1:**
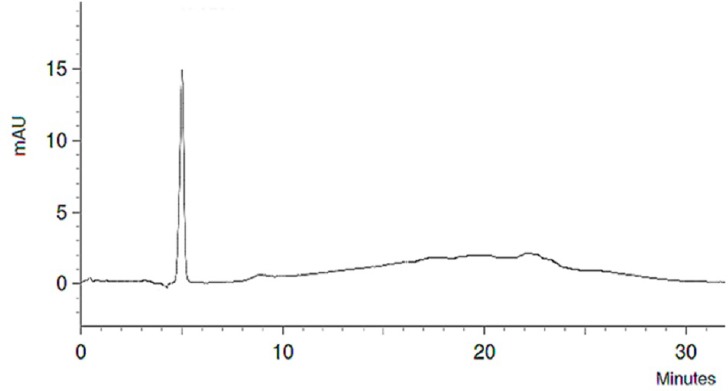
HPLC chromatogram of MC-LR standard solution-1: microcystin-LR

Despite no TFA existed in the used solvents, this method had a good sensitivity and high resolution in separation of MC-LR compound. The limit of detection in this method was 0.02 µg/mL, so this method is very sensitive and suitable for MC-LR detection in aqueous samples. The analytical chromatogram of MC-LR is shown in Figure 1. As shown in this figure MC-LR has been detected in 5.33 minute with suitable resolution.

## 4. Discussion

MC-LR was detected in short retention time with appropriate sensitivity and high resolution without existence of any acidic organic modifier in mixture of solvents. Many investigations reported different retention times and resolutions compared to present study in the analysis of MCs with reversed phase chromatography. Some of the major differences between other studies and present study are in the percentage and combination of organic solvent and silanol activity of stationary phase.

### 4.1 Effect of Organic Solvent Percentage

MC-LR is a hydrophilic variant of MCs, so polar solvents are suitable for elution of MC-LR molecules in reversed phase chromatography. In the analysis of hydrophilic molecules with reversed phase chromatography, decreasing of organic solvent percentage against water percentage (not less than 10%) decreases the elution time. On the other hand, increasing the organic solvent percentage can result in increased retention time. So, percentage of organic solvent is effective in achieving retention time of sample molecules (Mayer, 2004).

Many investigations have used several mixture of water and organic solvents (such as methanol-water and acetonitrile-water) with various percentages for the analysis of MC-LR, so, they have achieved several retention times and resolutions (Purdie et al., 2009). Selected percentage of acetonitrile (ACN) and water in used gradient method in the present study could achieve shorter retention time than other investigations and also achieving suitable sensitivity and resolution. Application of methanolic solvents as the mobile phase can cause some disadvantages. *Purdie* has reported using methanolic solvent in MC-LR molecules elution can cause in decreased resolution and increased retention time (Purdie et al., 2009). In addition, generally elution of MC-LR methanolic solvents have minor recovery than ACN solvents (Pyo, Dongjin, & Hyundu Shin, 2002). *Rapala* has reported decreasing HPLC methods sensitivity by increasing methanol concentration in samples (Rapala et al., 2002). Another negative effect of using methanol in mobile phase is higher viscosity of methanol solutions than other organic solvents in the same percentages (Mayer, 2004). It can cause more pressure drop over the column and thus rise in the required pressure. Therefore, many of the investigations preferred ACN solutions as mobile phase in different percentages, as a result they reported many different retention times and different sensitivities. In the present study, with using variable percentages of ACN (soluble in water) in the desired range (in gradient run) of MC-LR was detected in a shorter time than other studies and was achieved suitable resolution without any TFA in the mixture of mobile phase.

### 4.2 Silanol Activity of Stationary Phase

Another effective parameter on sensitivity and resolution of analysis method is silanol activity of stationary phase. Silanol groups are a functional group of silica surface which are considered having strong adsorption sites with hydrophilic nature. ODS (octadecylsilane) is non-polar and is a suitable choice for use in reversed phase chromatography, but there is a pH limited range for using silica-based stationary phases. At pH less than 2, siloxane bonds begin to hydrolyze and at pH higher than 8 silica particles can dissolve. Also, when silica is used with an alcohol, it can be converted to Esther. Esterified silica is disposed to hydrolysis, so it can’t be used with mobile phases, including water and alcohol. Several studies have reported different results in using different columns. *Rapala* reported a weak separation by using ammonium acetate and ACN as mobile phase and zorbax column, also no separation in analyzed MCs variant occurred when they used zorbax column with 0.05% TFA and ACN as the mobile phase. However, when these solvents were used by a column which had a stronger silanol activity, they reported a strong separation. Compared with the present study, the difference in flow rates, silanol activity of used column, and presence of TFA can be the cause for the differences in retention time and separation. *Aguete* determined MCs with 40% acetonitrile and 0.05% TFA (as acidic organic modifier) as mobile phase. Under the effect of TFA, they achieved good separation but longer retention time than present study which could be as a matter of greater percentage of ACN, also the method sensitivity was minor than the present study. *Barco* detected MC-LR by using 0.05% TFA and ACN as mobile phase. They reported a 17 min retention time for MC-LR (Barco et al., 2005). Also, they used TFA as acidic organic modifier but silanol activity of stationary phase was lower than the present study.

## 5. Conclusion

Good separation and high resolution in MC-LR analysis was achieved by choosing a suitable percentage of solvents (ACN and water) and using a column with high silanol activity without the need to add TFA. Despite any acidic organic modifier in the mixture of mobile phase, acceptable separation and high resolution was achieved in Microcystin-LR analysis. This method is suitable for laboratory purposes and determination of MC-LR in laboratory synthetic samples and is suitable for MC-LR detection in water samples. Worth to add, that all the devices should be accurately cleaned up. During performing this method, pH level was kept constant in 7.6. Therefore, we suggest that other investigations be performed with variable pH levels to determine the effect of pH on separation, retention time, and LODs. Overall this method is economically a cost benefit method and it is a fast method because of low consumption of acetonitrile as mobile phase.
